# The linkage between medical student readiness for interprofessional learning and interest in community medicine

**DOI:** 10.5116/ijme.5f89.83ae

**Published:** 2020-11-06

**Authors:** Yusuke Matsuzaka, Yuko Hamaguchi, Ayako Nishino, Kumiko Muta, Ikuko Sagara, Hiroyuki Ishii, Ikue Noguchi, Sayaka Kuba, Yuji Shiotani, Takashi Mine, Tatsuki Ichikawa, Hiroki Ozawa, Toru Yasutake, Alan Kawarai Lefor, Sumihisa Honda, Takahiro Maeda, Yasuhiro Nagata

**Affiliations:** 1 Center for Comprehensive Community Care Education, Nagasaki University Graduate School of Biomedical Sciences; 2 Department of Comprehensive Community Care Service, Nagasaki Junshin Catholic University; 3 Department of Comprehensive Community Care Systems, Nagasaki University Graduate School of Biomedical Sciences; 4 Department of Neuropsychiatry, Nagasaki University Graduate School of Biomedical Sciences; 5 Advanced Medical Education Center, School of Medicine, Nagasaki University; 6 Department of Surgery, Jichi Medical University; 7 Department of Nursing, Nagasaki University Graduate School of Biomedical Sciences; 8 Department of Community Medicine, Nagasaki University Graduate School of Biomedical Sciences

**Keywords:** Interprofessional learning, community medicine, medical education, undergraduate curriculum

## Abstract

**Objectives:**

The purpose of this study is to investigate the relationship between medical student readiness for interprofessional learning and interest in community medicine prior to incorporating community-oriented interprofessional education into the curriculum.

**Methods:**

A questionnaire was administered to students at Nagasaki University School of Medicine in Japan during each of three consecutive years (N=2244). The Readiness for Interprofessional Learning Scale (RIPLS) was administered in addition to a questionnaire to evaluate interest in community medicine. The Kruskal-Wallis and Steel-Dwass tests were used to determine differences between school years. Correlation between the RIPLS score and interest in community medicine was evaluated with Spearman's rank correlation coefficient. Relationships between RIPLS score and demographic parameters, and interest in community medicine were evaluated with multiple linear regression analysis.

**Results:**

Eighty-four percent (1891/2244) of students responded. The RIPLS score was highest in school year 1, followed by year 6, year 5, year 3, and years 4 and 2. Interest in community medicine correlated with the RIPLS score (r_s_ = 0.332, p < 0.001), but less in year 1 (r_s_ = 0.125, p = 0.002) than in other years. RIPLS score was significantly associated with gender, age, school year, interest in community medicine, but not the year that the survey was conducted.

**Conclusions:**

Community-oriented interprofessional education has the potential to improve attitudes towards interprofessional learning. When introducing this promising education into the curriculum from year 1, attracting students' interest in community medicine should be considered.

## Introduction

Interprofessional collaboration is essential for healthcare systems due to the recent global shift from the traditional patient-doctor relationship to collaborative and team-oriented approaches to patient care.^1^ To enhance collaboration in future health care systems, it is necessary to increase exposure to formalized interprofessional education (IPE) across health professions during training. By definition, IPE is said to occur "when two or more professions learn with, from and about each other to improve collaboration and the quality of care".^2^ While interprofessional collaboration is an expected competency, many undergraduate healthcare educational programs lack adequate IPE experiences.^3^ More recently, medical schools around the world have been incorporating it in various ways in their curricula.^4^ However, questions remain about how and when is best to educate and train students for interprofessional collaboration.

We are focusing on community-based medical education as an opportunity to uncover the potential of interprofessional collaboration. Since medical students can obtain the necessary knowledge, skills, and attitudes for medical procedures in community health care, this environment offers many opportunities for interprofessional learning.^5^^-^^8^

In order to incorporate community-oriented IPE into the curriculum for medical education, the degree of preparation of a medical student's interprofessional learning in all school years must first be clarified. The Readiness for Interprofessional Learning Scale (RIPLS), developed by Parsell and Bligh,^9^ was adapted in this study and has been broadly adopted to evaluate attitudes among healthcare students toward interprofessional collaboration.^10^^-^^13^ Students' attitudes toward interprofessional learning seem to be affected by multiple factors, such as generation atmosphere, current curriculum, social factors, and so on. Therefore, it is not appropriate to assess their attitudes in a single one year-survey.^3^^,^^14^^,^^15^ To avoid bias due to a single year, and students were surveyed annually for three years. Differences in their attitudes in each school year as they progress through the medical curriculum should be clarified by a repeated cross-sectional study.

This study aims to investigate the readiness of medical students for interprofessional learning over three academic years and the relationship between students' attitudes toward interprofessional learning and their interest in community medicine prior to incorporating community-oriented interprofessional education into the curriculum.

## Methods

### Study design

A cross-sectional study was performed among students at the Nagasaki University School of Medicine in Japan by survey in each of three academic years (2015-2017). The Japanese version of the Readiness for Interprofessional Learning Scale (RIPLS) was administered, which has sufficient reliability, as confirmed by translation and back-translation.^16^ This scale includes 19 items, each of which is assessed on a five-point Likert scale: 1 (strongly disagree) to 5 (strongly agree). In addition to the RIPLS, we prepared a questionnaire to evaluate student interest in community medicine. This questionnaire also used a five-point Likert scale: 1 (not interested) to 5 (very interested). This study was performed at the same time each year. The students were informed both orally and in a written leaflet about this survey and invited to complete the questionnaire. Survey responses were confidential, and personal information was removed. This study was approved by the ethical review board of Nagasaki University.

### Participants

A total of 2244 medical students at Nagasaki University were invited to participate in this study over three years, and 1891 students (84.3%) agreed. There were 534 (28.2%) female students. The characteristics of the participants is shown in Table 1. Most students in the Nagasaki University School of Medicine are admitted directly from high school and complete a standard six-year curriculum, culminating in graduation with the equivalent of a Doctor of Medicine degree.

**Table 1:** Participant characteristics

Characteristics		
Number of students		2244
Number of participants		1891
Response rate (%)		84.3
Age (Average)		22.7
Standard Deviation		3.66
Gender		
	Male	1330
	Female	534
	Unknown	27
School Year		
	Year 1	350
	Year 2	330
	Year 3	325
	Year 4	317
	Year 5	311
	Year 6	231
	Unknown	27

### Statistical analysis

The average RIPLS score for each student was assessed. The median of the average scores for each school year were compared using the Kruskal-Wallis and Steel-Dwass tests. Correlation between the RIPLS score and interest in community medicine was evaluated by Spearman's rank correlation coefficient as "r_s_". Using multiple linear regression analysis, the relationships between the RIPLS score and school year, gender, age and the year the survey was conducted, and interests in community medicine were evaluated. A correlation coefficient of 0.1 is a weak association; a correlation coefficient of 0.3 is considered a moderate correlation, and a correlation coefficient of 0.5 or higher is deemed a strong correlation. The Kruskal-Wallis and Steel-Dwass tests were performed using EZR software. The other tests were performed using IBM SPSS Statistics version 26.0 for Windows (IBM, Armonk, NY, USA).

## Results

### RIPLS score among each school year

The median RIPLS scores are shown in Table 2. The scores of female students were significantly higher than those of males (U (N_male_ = 1330, N_female_= 534) = 411830, z = 5.402, p < 0.001).There is a statistically significant difference in the RIPLS score among each school year (H(5)=181.21, p< 0.001). The differences in the RIPLS score between each school year by post-hoc Steel-Dwass test are shown in Table 3.

**Table 2:** Median RIPLS score in each year

Variables	Median	Interquartile Range
All respondents	4.00	3.68-4.42
Gender		
Male	3.95	3.63-4.37
Female	4.05	3.79-4.58
School Year		
Year 1	4.32	3.95-4.63
Year 2	3.84	3.47-4.16
Year 3	3.89	3.47-4.21
Year 4	3.84	3.47-4.13
Year 5	4.00	3.74-4.53
Year 6	4.05	3.79-4.58

RIPLS: Readiness for Interprofessional Learning Scale

**Table 3:** P-values comparing RIPLS scores by school year*

Year	Year 1	Year 2	Year 3	Year 4	Year 5	Year 6
Year 1	-	< 0.001	< 0.001	< 0.001	< 0.001	0.004
Year 2		-	0.968	0.999	< 0.001	< 0.001
Year 3			-	0.808	< 0.001	< 0.001
Year 4				-	< 0.001	< 0.001
Year 5					-	0.864

RIPLS: Readiness for Interprofessional Learning Scale *p-values calculated by Steel-Dwass test

### Interest in community medicine and correlation with RIPLS

The average scores of interest in community medicine in each school year and their correlation with RIPLS are shown in Table 4. The interest in community medicine at year 1 was highest, but its correlation with the RIPLS score (r_s_ = 0.125, p = 0.002) was weaker than that in other school years.

### Multiple linear regression analysis

As shown in Table 5, the RIPLS score was significantly related in the following order: standardized regression coefficient, gender, age, school year (Year), and interest in community medicine. The RIPLS score was not significantly related to the academic year that the survey was conducted.

**Table 4:** Interest in community medicine in each school year and correlation with RIPLS

School Year	Mean	SD	r_s_	*p*-value
Year 1	3.99	1.07	0.125	0.022
Year 2	3.68	1.05	0.377	< 0.001
Year 3	3.69	1.00	0.328	< 0.001
Year 4	3.61	1.09	0.460	< 0.001
Year 5	3.77	0.88	0.228	0.002
Year 6	3.76	0.92	0.347	< 0.001

RIPLS: Readiness for Interprofessional Learning Scale, SD: Standard deviation

## Discussion

There is no doubt that physicians must have the ability to collaborate with other professionals as members of the health care team. For that reason, IPE is required for medical students, and is required to be incorporated into the curriculum for medical education. In this study, a survey was given annually for three years to investigate the medical students' readiness for interprofessional learning and the relationship between students' attitude toward interprofessional learning and their interest in community medicine. Most previous studies have compared RIPLS scores before and after teaching a specific curriculum and shown an increased RIPLS after IPE. The present study was not a survey of short-term results before and after a special program, but a longitudinal survey across all school years. A student's attitude toward interprofessional learning can be influenced by several factors, so a one-year survey alone may reflect a student's transitory trends. In this study, to avoid bias due to a single year, three academic years of data were collected and analyzed together.

We found that the RIPLS score of students in year 1 was highest, followed by year 6, year 5, year 3, year 4, and year 2. Immediately after admission to medical school, students tend to be open-minded to various disciplines. This mindset in year 1 may partially explain the positive attitude towards interprofessional learning. A previous report has shown a decline in RIPLS scores after IPE from medical school matriculation.^17^ It has been maintained that IPE may reinforce negative beliefs about the value of learning from other health professionals.^17^ Research has found that students in various healthcare professions enter the university with stereotypical views of one another.^18^ For medical students, especially undergraduates, year 1 involves transitioning into adulthood and dealing with professional identity formation.^19^ This may be the reason why the RIPLS score decline in year 2. However, it is reported that RIPLS scores partially increase during the curriculum.^20^ In our study at Nagasaki University, expectations for interprofessional collaboration are believed to have increased since year 2 by adopting the IPE program

**Table 5:** Multiple linear regression analysis (dependent variable, RIPLS score)

Variables	Regression Coefficient	Standard error	Beta	*t*	*p*-value
Constant definition	3.412	0.112		30.383	< 0.001
Year 2^*^	-0.503	0.050	–0.309	-10.149	< 0.001
Year 3^*^	-0.365	0.050	–0.225	-7.274	< 0.001
Year 4^*^	-0.395	0.049	–0.259	-8.004	< 0.001
Year 5^*^	-0.262	0.053	–0.168	-4.937	< 0.001
Year 6^*^	-0.213	0.050	–0.148	-4.234	< 0.001
Gender (reference = male)	0.183	0.031	0.150	5.864	< 0.001
Age	0.017	0.005	0.114	3.645	< 0.001
Interest in community medicine	0.118	0.014	0.216	8.410	< 0.001
Academic year 2016^**^	-0.005	0.033	-0.005	-0.156	0.876
Academic year 2017^**^	0.016	0.043	0.013	0.382	0.703

RIPLS: Readiness for Learning Scale; *reference = Year 1, **reference = Year 2015

from the lower school year. In addition to the above, participation in clinical training from year 4 may be another reason. This study showed that the RIPLS score among medical students correlates with student interest in community medicine. Students at Nagasaki University could imagine community-based medical care and comprehensive medical care on remote islands as community medicine. The change in interest in community medicine in each school year parallels the RIPLS score. However, statistically, the correlation between them is moderate in year 4 and above.

Walker and colleagues determined that there is a greater opportunity to collaborate with other professionals in community settings.^21^ Community-based clinical learning environments constitute a rich resource, whereby students can create constructive and transformative interprofessional learning experiences. In that way, students can learn the importance of collaborating with other health professionals in community-based medical education.^22^ Reinforcing our IPE program in a rural setting could also have an impact on students with lower interest in interprofessional learning.

The present study has several limitations. First, there is a social desirability bias. Students in the first year tend to answer questions in a manner that will be viewed favorably by others. Second, we conducted this study at a single institution. However, our student population is overall representative of most medical school populations because we follow the model core curriculum in Japan.

The curricula of medical schools are constantly being updated. It may be difficult to examine how a particular educational program could affect the attitudes of medical students with all curricula. Successive RIPLS observations could be used to assess the effectiveness of IPE throughout the curriculum. IPE programs should be adjusted to meet the demands of different students in different years of medical education and should help enhance future interprofessional collaboration.

## Conclusions

To increase interest in community medicine, medical schools should consider the introduction of community-oriented interprofessional education into the curriculum in year 1. Community-based medical education has the potential to improve attitudes towards interprofessional cooperation in the future, which will hopefully contribute to improved patient care and increased professional satisfaction for all members of the health care team.

### Acknowledgments

We thank the medical students who participated in our study.

### Conflict of Interest

The authors declare that they have no conflict of interest.
